# The Hospital Burden of Flu in Italy: a retrospective study on administrative data from season 2014–2015 to 2018–2019

**DOI:** 10.1186/s12879-024-09446-2

**Published:** 2024-06-08

**Authors:** Riccardo Cipelli, Serena Falato, Eleonora Lusito, Giovanni Maifredi, Michele Montedoro, Paola Valpondi, Alberto Zucchi, Maria Vittoria Azzi, Laura Zanetta, Maria Rosaria Gualano, Entela Xoxi, Paola Giovanna Marchisio, Silvana Castaldi

**Affiliations:** 1 IQVIA Solutions Italy Srl, Via Fabio Filzi 29, Milan, 20124 Italy; 2 SS Epidemiologia, Agenzia di Tutela della Salute di Brescia, Brescia, Italy; 3 UVARP ASL Foggia, San Severo c/o Ospedale, Italy; 4 ULSS 8 Berica, Vicenza, Italy; 5 UOC Servizio Epidemiologia presso ATS di Bergamo, Bergamo, Italy; 6 Sanofi SpA, Milan, Italy; 7 Saint Camillus International University of Health Sciences, Rome, Italy; 8 Università Cattolica del Sacro Cuore, Alta Scuola di Economia e Management dei Sistemi Sanitari (ALTEMS), Rome, Italy; 9 Dipartimento di Fisiopatologia Medico-Chirurgica e dei Trapianti, University of Milan, Milan, Italy; 10 Department of Biomedical Sciences for Health, University of Milan, Milan, Italy; 11 Fondazione IRCCS Ca’ Granda Ospedale Maggiore Policlinico, Milan, Italy

**Keywords:** Influenza, Hospitalization, Burden, Costs, Comorbidities, Complications, Administrative database, Local health units, Secondary health care

## Abstract

**Background:**

Every year in Italy, influenza affects about 4 million people. Almost 5% of them are hospitalised. During peak illness, enormous pressure is placed on healthcare and economic systems. This study aims to quantify the clinical and economic burden of severe influenza during 5 epidemic seasons (2014–2019) from administrative claims data.

**Methods:**

Patients hospitalized with a diagnosis of influenza between October 2014, and April 2019, were analyzed. Clinical characteristics and administrative information were retrieved from health-related Administrative Databases (ADs) of 4 Italian Local Health Units (LHUs). The date of first admission was set as the Index Date (ID). A follow-up period of six months after ID was considered to account for complications and re-hospitalizations, while a lookback period (2 years before ID) was set to assess the prevalence of underlying comorbidities.

**Results:**

Out of 2,333 patients with severe influenza, 44.1% were adults ≥ 65, and 25.6% young individuals aged 0–17. 46.8% had comorbidities (i.e., were at risk), mainly cardiovascular and metabolic diseases (45.3%), and chronic conditions (24.7%). The highest hospitalization rates were among the elderly (≥ 75) and the young individuals (0–17), and were 37.6 and 19.5/100,000 inhabitants/year, respectively. The average hospital stay was 8 days (IQR: 14 − 4). It was higher for older individuals (≥ 65 years, 11 days, [17 − 6]) and for those with comorbidities (9 days, [16 − 6]), p-value < 0.001. Similarly, mortality was higher in elderly and those at risk (p-value < 0.001). Respiratory complications occurred in 12.7% of patients, and cardiovascular disorders in 5.9%. Total influenza-related costs were €9.7 million with hospitalization accounting for 95% of them. 47.3% of hospitalization costs were associated with individuals ≥ 65 and 52.9% with patients at risk. The average hospitalisation cost per patient was € 4,007.

**Conclusions:**

This retrospective study showed that during the 2014–2019 influenza seasons in Italy, individuals of extreme ages and those with pre-existing medical conditions, were more likely to be hospitalized with severe influenza. Together with complications and ageing, they worsen patient’s outcome and may lead to a prolonged hospitalization, thus increasing healthcare utilization and costs. Our data generate real-world evidence on the burden of influenza, useful to inform public health decision-making.

**Supplementary Information:**

The online version contains supplementary material available at 10.1186/s12879-024-09446-2.

## Background

Influenza is an acute viral respiratory infectious disease which occurs worldwide with a mostly seasonal frequency [1]. Incidence, onset of the epidemic season, and relative duration are characterized by high variability in Italy. Overall, influenza affects on average 9% of the Italian population each year, with a minimum of 4%, observed in the 2005-06 season, and a maximum of 15% recorded in the 2017-18 [2]. Epidemiological differences can also be observed according to the age of individuals, and virus strain. Influenza types/subtypes A (H1N1) and A (H3N2) are more frequent among young children and the elderly respectively, while the subtype B spreads mostly among older children [3–5]. Influenza epidemics can cause overcrowding of clinics and hospitals during peak illness periods, high levels of worker/school absenteeism and productivity losses. Although most people recover from symptoms within a week without requiring medical attention, influenza may degenerate into severe conditions, hospitalizations, or death especially in people with pre-existing comorbidities and in vulnerable individuals like the younger and the older. Complications may also arise and lead to a bad prognosis. Among others, respiratory complications, viral or bacterial superinfections (pneumonia), or the worsening of underlying chronic conditions are the most common illnesses (5–6). About 290,000 to 650,000 deaths from respiratory causes alone are attributable to influenza each year [7]. Extra-respiratory complications such as cardiovascular and nervous system complications [8, 9] also play a relevant role. Epidemiological studies [10, 11] have demonstrated an association between influenza epidemics and cardiovascular mortality which is decreased in adults undergoing influenza vaccination, the most effective medical intervention to prevent infection. Quantifying the clinical and economic burden associated to patients hospitalized with influenza in terms of healthcare resource utilization and direct medical costs is therefore important for decision makers to optimize influenza prevention policies. This real-world evidence study aims to assess healthcare resource consumption and direct medical costs using administrative data (AD) from four Local Health Units (LHUs) in Italy.

## Methods

### Data source

The study was conducted using anonymized claims data from AD of four LHUs (Agenzia di Tutela della Salute (ATS) Brescia, Agenzia di Tutela della Salute (ATS) Bergamo, Azienda Unità Locale Socio-Sanitaria (ULSS) 8 Berica, and Azienda Sanitaria Locale (ASL) Foggia) recording data about hospitalizations and covering reimbursement information for each public or private hospital. LHUs were selected based on the following criteria: (i) data quality; (ii) ethics committee sessions regularly held; (iii) geographical distribution across the country (i.e., each LHU was picked to represent different areas); (iv) large catchment area. Overall, they included approximately 3,300,000 health-assisted individuals. Each database holds the following data:

- patient demographic data including the year of birth, the gender, and the exemption code (if any).
- hospitalizations recorded through the Hospital Discharge Records (SDO) which include admission/discharge dates, type of hospitalization (ordinary or day hospital), main and secondary diagnosis coded according to the International Classification of Diseases, Ninth Revision, Clinical Modification (ICD-9-CM), Diagnosis-Related Group (DRG) reimbursement rate, information on transfer between wards and destination after hospitalization, the exemption code (if any) and the costs.
- outpatient care which includes specialist visits, medical procedures and exams with dates, exemption code (if any) and costs.
- Emergency Room (ER) accesses with their relative date, main and secondary diagnoses (coded with ICD-9), treatment received including the pharmacological one, type of exams completed and costs.
- prescribed drugs from hospitals and pharmacies in the area with the date of prescription, the prescribing physician’s number, and the Anatomical-Therapeutic-Chemical (ATC) code of the prescribed drug, the exemption code (if any) and the costs.

Information extracted by each LHU was organized in five databases (DB): (1) Beneficiaries’ DB containing demographic data; (2) Hospitalization DB including information on hospitalizations; (3) Diagnostic tests Laboratory DB, containing data on laboratory tests, and specialist visits DB with information on outpatient specialist services; (4) ER DB containing information on patients’ access to ER, and (5) Pharmaceuticals DB containing information on medication prescriptions.

Patient’s information was anonymized in full compliance with the Italian code of protection of personal data (Legislative Decree, 101/18 and 196/03). An anonymous and univocal numerical patient identifier was assigned to each participant to guarantee patients’ privacy. All the results of the analysis were produced as aggregated summaries to avoid any direct or indirect link to individual patients.

### Cohort definition

**Fig. 1:**
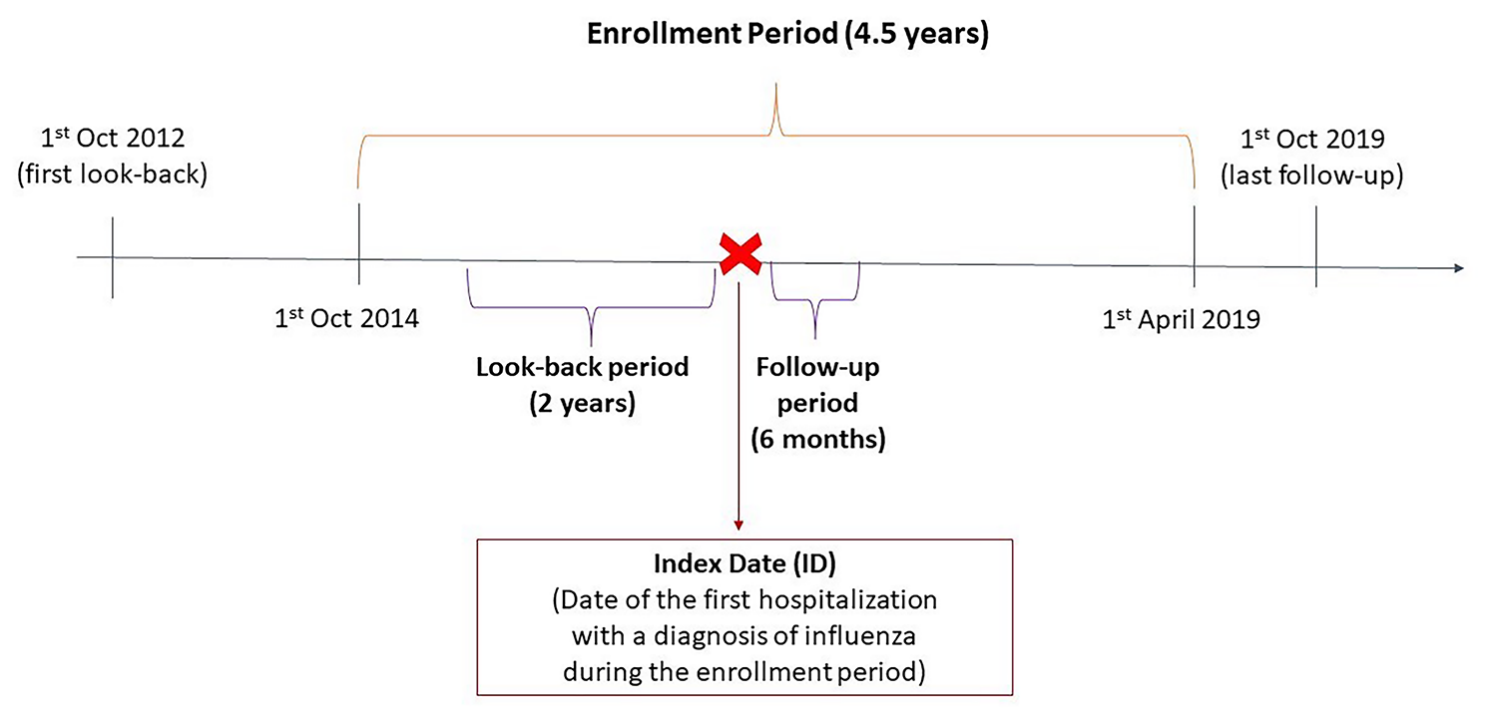
The schematic study design. The study design is shown above. The study period spanned from the 1st October 2014 to 1st April 2019: during this period, any patient hospitalized with a diagnosis of influenza was identified. The study Index Date (ID) corresponded to the first hospitalization due to influenza. From the ID, a 6-months follow-up period was considered for each patient, to account for mortality, re-hospitalization, healthcare resource consumption, direct healthcare costs, and influenza related complications (e.g.: for patients enrolled on 1st April 2019 the follow-up period ended on 1st October 2019). A look-back period of 24 months was considered for each patient to account for comorbidities

This was a retrospective, non-interventional descriptive study to examine the clinical and economic burden of patients with severe seasonal influenza who required hospital admission. The study period spanned from 1 October 2014 to 1 April 2019 for a total of 5 years and 5 epidemic seasons (Fig. 1). The end of the study was set to 2019 to prevent from the bias due to COVID-pandemic that caused a substantial reduction of influenza transmission. Diagnosis explicitly associated with an influenza virus like influenza with pneumonia, with other (respiratory) manifestations and due to identified influenza viruses were considered and extracted from hospital admissions, and ER access looking at both primary and secondary diagnosis. The following ICD-9 codes were used, based on the literature [12, 13]: 487.0, 487.1, 487.8 and 488. Patients who relocated outside the LHU during the observation period, i.e., in the 6 months of follow-up were excluded. The date corresponding to the first hospitalization with a diagnosis of influenza during the observation period was defined as “Index Date” (ID). Each patient was followed for 6 months after ID according to previous similar research [14] and back for 2 years in the look-back period to evaluate the presence of comorbidities (Fig. 1). Overall, the observation period spanned from 1 October 2012 to 1 October 2019.

### Information extracted from the databases

To provide demographic characteristics of the study population, information regarding age and sex was extracted for each patient at the time of initial diagnosis. Clinical characteristics consisting in the presence of comorbidities, were identified looking at all ICD-9 or exemption codes for chronic diseases, recorded during the 24-month period before ID. The ones that, based on the literature [15–18] worsen the clinical outcome and hence defined the risk groups were: diabetes, immune deficiency syndromes, chronic rheumatic heart disease, ischemic heart disease, disease of pulmonary circulation, other forms of heart disease, chronic kidney disease, chronic liver disease, neoplasms, chronic respiratory disease, pregnancy, preterm birth, haemoglobinopathy, metabolic disorders, cystic fibrosis, neuromuscular disorders, neurologic disorders and genetic syndromes. Comorbidities were extracted from SDO and ER access. Morbidities which identify complications observed in patients hospitalized with severe influenza were also considered and searched in the 6 months follow-up period. They were selected based on the available literature [15, 18–25] and were: respiratory complications, cardiovascular, neurological, and diabetic complications, and co- and secondary infections. Exemption codes were not used to identify complications from influenza. Patients were categorized based on the number of comorbidities of interest. A patient without any comorbidity was classified as “not at risk”.

### Study outcomes

The main outcomes considered in the analysis were: monthly number of hospitalizations, healthcare resource utilization (HRU) and direct healthcare costs. The last two outcomes referred to pharmacological treatments (number of prescribed drug-packs and days of treatment), outpatient visits (number), diagnostic procedures and laboratory tests (number), ER accesses (number of admissions, treatments, and exams), day hospital (number) and inpatient episodes (number and length of stay). Number of hospitalizations, resource consumption and costs were stratified by age categories (0–17, 18–49, 50–59, 60–64, 65–74, ≥ 75) and risk groups. Total healthcare direct costs were estimated considering the costs associated to all mentioned items. Additionally, all cause-in-hospital mortality following influenza hospitalization, the occurrence of complications, the hospitalization rates based on the total population in the LHU catchment area and the number of all cause re-hospitalizations of influenza cases, all stratified by sex, age category, and risk group, were estimated, and evaluated.

### Cost analysis

Drug costs were evaluated using the Italian National Health Service (INHS) purchase price. Hospitalization costs were provided by the LHUs and reported in the SDO. The mean annual health care costs per patient were evaluated based on total resource consumption (considering drugs, hospitalizations, outpatient visits and inpatients episodes, ER access, day hospital, tests and laboratory exams) during the follow-up period. Costs reported in the SDO represent the reimbursement levels of the NHS to health care providers. The outpatient service costs were defined according to the tariffs applied by the evaluated regions.

### Statistical analysis

All analysis were performed with SAS® software 9.4 or later and were descriptive. Categorical variables were expressed as numbers (N) and percentages (%), whereas quantitative variables through mean ± SD. The 95% confidence intervals (CIs) for proportions and means were calculated using the Wald confidence interval formula. The non-parametric Mann-Whitney U-Test was used to compare outcomes between two independent groups. The non-parametric Kruskal-Wallis test was used to compare two or more groups for continuous or discrete variables.

### Data management and quality control

Data used for the study were collected in the LHU data warehouse. These data are sent every month by the LHUs to the Region of reference, with a delay specific for each data flow. These administrative data are collected for reimbursement purposes and their completeness is implicitly guaranteed by the LHU. After study approval by the LHU and Ethics Committee, LHU staff was requested to extract the data. A process of quality control of data was performed by the LHUs through which the internal consistency of data was verified to ensure the completeness and reliability of them. Given the nature of real-world data, and due to variabilities in efficiency and completeness of records, missing data are likely to be present. No attempt to input missing data was made.

## Results

### Demographic and clinical characteristics of patients hospitalized with influenza in Italy

Table 1 shows demographic and clinical characteristics of patients hospitalized with a diagnosis of influenza in the Italian LHUs over the whole study period (1 October 2014–1 April 2019). A total of 2,333 patients met the study inclusion criteria. Of these, 1,190 (51%) were males and 1,143 (49%) were females. On average, individuals were aged 50 years (95% CI, 48.7–51.3)Approximately 80% of influenza-related hospitalizations occurred in individuals at extreme age categories (p-value < 0.05), i.e., young adults aged 0–17 (*N* = 596; 25.6%, 95% CI, 23.8–27.3),of these 68.3% (*N* = 407) were infants and children aged 0–4, and in adults aged ≥ 65 (*N* = 1,029; 44.1%, 95% CI, 42.1–46.1). More than half of the enrolled patients (*N* = 1,240; 53.2%, 95% CI, 51.1–55.1) had no comorbidities. A diagnosis of one comorbidity was observed in 558 patients (23.9%), whilst two comorbidities were recorded in 535 patients (22.9%). The group of patients not at risk was mainly composed of young patients aged 0–17 while the group of patients at risk was composed by elderly individuals. Among the comorbidities, cardiovascular and metabolic diseases were the most prevalent (*N* = 1,056; 45.3%) followed by chronic medical conditions (*N* = 577; 24.7%), neoplasms (*N* = 398; 17.1%) and disorders of pulmonary circulation (*N* = 234; 10.0%).

**Table 1:** Demographic and Clinical characteristics of patients hospitalized with influenza referred to the entire study period

	Patients hospitalized with influenza (*N* = 2,333)	
	*N*	%
**Sex**		
*Male*	1,190	51.0
*Female*	1,143	49.0
**Age (years)**		
*Mean (SD)*	50 (31.6)	
*Median*	59	
**Age - group (years)**		
*0–17*	596	25.6
*18–49*	378	16.2
*50–59*	218	9.3
*60–64*	112	4.8
*65–74*	318	13.6
*≥75*	711	30.5
*≥85*	281	12.0
Comorbidities*		
*Diabetes*	321	13.8
*Immune deficiency syndromes*	9	0.4
*Chronic Rheumatic Heart Disease*	212	9.1
*Ischemic Heart Disease*	312	13.4
*Diseases of Pulmonary Circulation*	234	10.0
*Other Forms of Heart Disease*	423	18.1
*Chronic Kidney Disease*	105	4.5
*Chronic Liver Disease*	71	3.0
*Neoplasms*	398	17.1
*Chronic Respiratory Disease*	189	8.1
*Pregnancy*	51	2.2
*Preterm birth*	17	0.7
*Haemoglobinopathy*	3	0.1
*Metabolic disorders*	10	0.4
*Cystic fibrosis*	10	0.4
*Neuromuscular disorders*	6	0.3
*Neurologic disorders*	7	0.3
*Genetic syndromes*	9	0.4
**Risk group**		
*Not at risk*	1,240	53.2
*1 comorbidity*	558	23.9
*2 + comorbidities*	535	22.9

* All comorbidities recorded in the 24 months before ID; percentages refer to the full cohort of patients (*N* = 2,333); recorded comorbidities are not mutually exclusive

### Hospital burden of patients with influenza

**Fig. 2:**
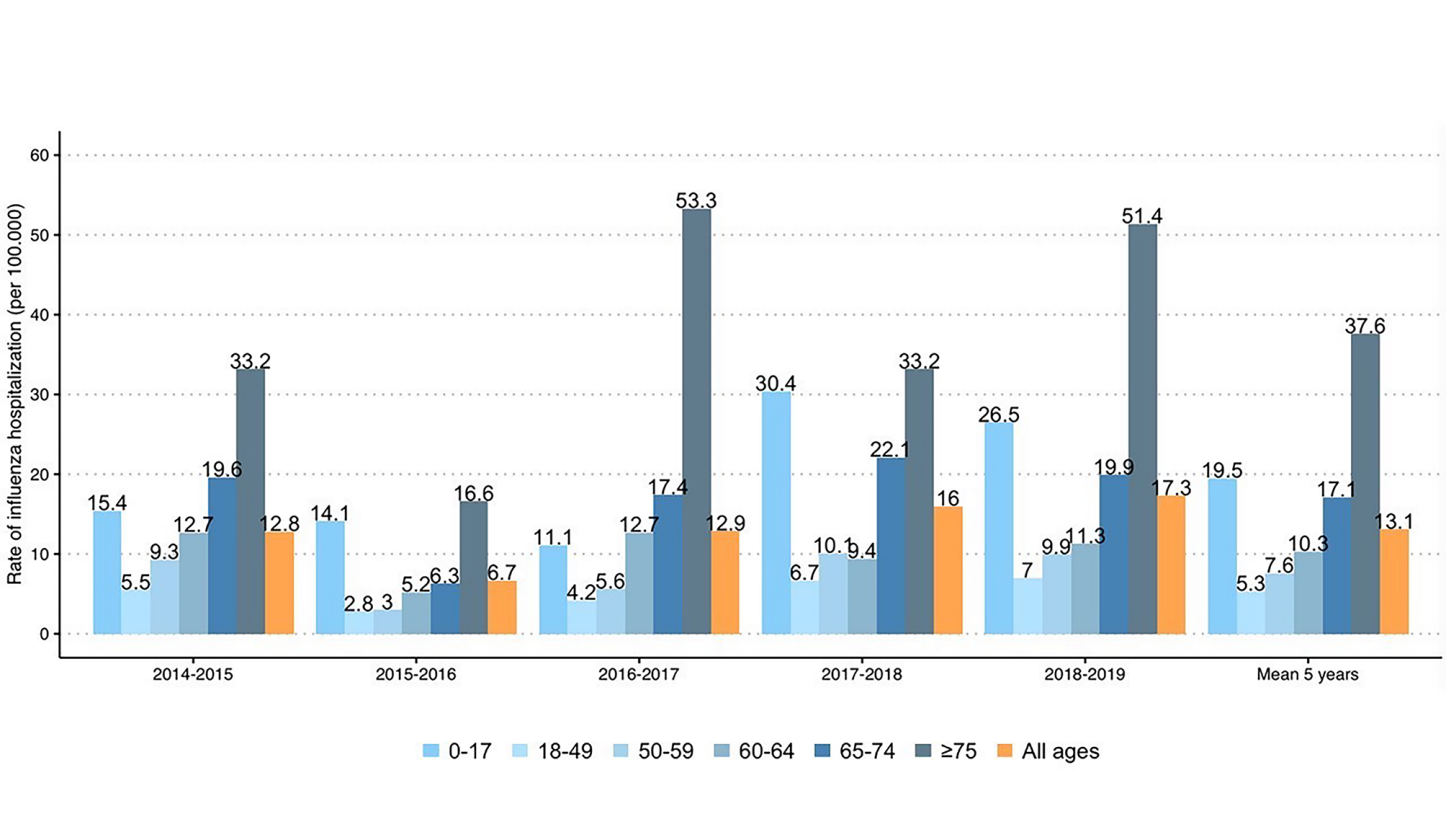
Hospitalization rates by influenza seasons stratified by age categories

Hospitalizations due to influenza (*N*), re-hospitalizations of influenza patients due to all-causes (*N*) and stay duration (mean (SD) and median (Q3-Q1)) are reported in Table 2 and refer to the whole study period or to the influenza season. Data are shown by age category and risk group. The study cohort (*N* = 2,333) contributed to a total of 2,349 hospitalizations, including re-admissions, recorded during the entire observation period. Over the five seasons 2,202 influenza hospitalizations were recorded. Forty-eight re-hospitalizations of influenza patients due to all causes were observed over the 5-year period. The rate of influenza hospitalizations varied among influenza seasons from 6.7/100.000 (inhabitants) in 2015–2016, to 16.0/100.000 and 17.3/100.000 in 2017–2018 and 2018–2019 respectively (Fig. 2 and Table S1), and reflected the national incidence of influenza infections and intensity [24]. The highest number of influenza hospital admissions and re-admissions during the whole study period, was observed among adults aged ≥ 75 (*N* = 718), and among individuals in the age category 0–17 (*N* = 604). The mean hospitalization rate over 5 years, was of 19.5 (95% CI, 18.7–56.4) and 37.6 (95% CI, 9.0–30.0) respectively (Fig. 2 and Table S1). The total number of hospitalizations and re-hospitalizations due to influenza over the whole observation period was comparable between high-risk and not at -risk patients (*N* = 1,107, and *N* = 1,242, p-value 0.525). The same was observed for the total hospitalizations due to influenza during each influenza season (Table 2). From ID to the end of the study period (1 April 2019), 48 patients were re-hospitalized due to influenza (Table 2). Almost 38% (*N* = 18) of them were < 17 years and 33% (*N* = 16) were ≥ 65. The median (Q3-Q1) inpatient length of stay (LOS) was of 8 days (14 − 4) and increased with age ≥ 65, *N* = 11 (17 − 6) and the presence of comorbidities, *N* = 9 (16 − 6). Overall, almost 39.0% (*N* = 919) of patients (*N* = 2,333) were managed by the general medicine department, 22.3% (*N* = 520) by the paediatrics department, 11.5% (*N* = 268) by the infectious and tropical diseases department. Around 8% (*N* = 177) required intensive care unit, adult or neonatal (Table S2). Of the 919 patients admitted to the general medicine department, 60.1% (*N* = 560) were at risk. Similarly, almost half (*N* = 128; 47.8%) adult patients hospitalized at the infectious and tropical diseases department had at least one comorbidity as well as patients hospitalized at the pneumology ward (*N* = 66, 60.0%). Only 74 young patients (14.2%) hospitalized at the paediatric department (*N* = 520) had comorbidities. Overall, most patients went home after discharge (*N* = 2,122; 91%; Table S3). Around 75% (*N* = 21) of patients institutionalized post hospital discharge (*N* = 28) were elderly (≥ 75). More than half of individuals transferred to another hospital or health facility (*N* = 127) were at risk (*N* = 73; 57.4%) and ≥ 75 (*N* = 57; 44.9%).

**Table 2:** Hospitalizations and length of stay of patients with influenza, by age categories and risk groups

	Age categories							Risk groups	
	All ages	0–17	18–49	50–59	60–64	65–74	≥ 75	Not at risk	At risk
**Hospitalizations due to influenza during the whole observation period, N**	2,349	604	374	218	109	326	718	1,242	1,107
**Hospitalizations due to influenza during influenza seasons, N**	2,202	554	340	200	106	312	690	1,145	1,057
*n, (%)*									
*2014-15*	426	86 (20.2)	71 (16.7)	50 (11.7)	26 (6.1)	71 (16.7)	122 (28.6)	220 (51.6)	206 (48.4)
*2015-16*	220	77 (35.0)	33 (15.0)	16 (7.3)	10 (4.5)	23 (10.5)	61 (27.7)	118 (56.6)	102 (46.4)
*2016-17*	434	64 (14.7)	55 (12.7)	29 (6.7)	26 (6.0)	64 (14.7)	196 (45.2)	185 (42.6)	249 (57.4)
*2017-18*	541	175 (32.3)	89 (16.5)	54 (10.0)	20 (3.7)	81 (15.0)	122 (22.6)	313 (57.9)	228 (42.1)
*2018-19*	581	152 (26.2)	92 (15.8)	51 (8.8)	24 (4.1)	73 (12.6)	189 (32.5)	309 (53.2)	272 (46.8)
**All cause re-hospitalizations**	48	18 (37.5)	8 (16.7)	6 (12.5)	0 (0.0)	9 (18.8)	7 (14.6)	25 (52.1)	23 (47.9)
**of influenza cases, N**									
**Length of stay (days)**									
*mean (SD)*	11.2 (13.4)	6.9 (8.1)	11.2 (23.6)	12.2 (11.9)	11.5 (9.6)	12.8 (12.4)	13.7 (9.9)	9.6 (11.3)	13 (15.2)
*median (Q3-Q1)*	8 (14 − 4)	5 (8 − 3)	5 (10 − 3)	8 (15 − 6)	8.5 (14 − 6)	9 (16 − 6)	11 (18 − 7)	6 (11 − 4)	9 (16 − 6)

All results are reported as absolute numbers (N) and percentages (%), in parenthesis. These last, are calculated over the total number of patients hospitalized during each influenza season by age categories and risk groups

### Healthcare resource consumption

Table 3 shows resource consumption referred to day hospitals and ER access during the follow-up time, outpatient visits, tests, and lab exams from the admission date to one week after discharge. ER accesses before hospitalization were 1,980 corresponding to 84.3%(95% CI, 82.9–85.8) of 2,349 total admissions due to influenza during the entire period. The highest number of ER visits was reported for elderly patients (age ≥ 75; *N* = 616; 31.1%, 95% CI, 29.1–33.1) and for young individuals (age 0–17; *N* = 538; 27.2%, (95% CI, 25.2–29.1)). On average, 1.3 (SD = 4.9) tests or lab exams per-patient were recorded from the hospital admission date (ID) to one week after discharge. Patients with at least one comorbidity, perform more diagnostic and laboratory exams (*N* = 1,787; 57.7%) than individuals without comorbidities (*N* = 1,309; 42.3%), *p* < 0.001. Similarly, the number of tests was higher in patients aged ≥ 75 (*N* = 966; 31.2%) than in patients in the other age categories. Day hospital was infrequent. Only 32 events were recorded overall. They were observed in individuals aged 0–17 (*N* = 10; 31.3%) and 18–49 (*N* = 12; 37.5%). A total of 275 outpatient consultations were recorded. Most of them were done by patients in the age category 0–17 (*N* = 86; 31.3%, 95% CI, 25.8–36.8) and ≥ 75 (*N* = 74; 26.9%, 95% CI, 21.6–32.1). The number of visits was higher in patients at risk (*N* = 154; 56.0%) and particularly in those with one pre-existing comorbidity (*N* = 92; 33.5%) compared to patients not at risk (*N* = 121, 44.0%), p-value 0.031.

**Table 3:** HRU by age categories and risk groups

Age categories (years)	Statistics	Day Hospital*	Outpatient visits**	ER accesses^2^ *	Tests and lab exams**
***All ages*** *(* *N = 2,333)*	N	32	275	1,980	3,096
	Mean (SD)	0.0 (0.1)	0.1 (0.4)	0.9 (0.4)	1.3 (4.9)
***0–17*** *(* *N = 596)*	N (%)	10 (31.3)	86 (31.3)	538 (27.2)	595 (19.2)
	Mean (SD)	0.0 (0.1)	0.1 (0.5)	0.9 (0.4)	1 (3.9)
***18–49*** *(* *N = 378)*	N (%)	12 (37.5)	50 (18.2)	314 (15.9)	582 (18.8)
	Mean (SD)	0.0 (0.2)	0.1 (0.5)	0.8 (0.5)	1.5 (5.2)
***50–59*** *(* *N = 218)*	N (%)	6 (18.8)	26 (9.5)	170 (8.6)	583 (12.4)
	Mean (SD)	0.0 (0.2)	0.1 (0.4)	0.8 (0.5)	1.8 (5.7)
***60–64*** *(* *N = 112)*	N (%)	3 (9.4)	6 (2.2)	94 (4.8)	141 (4.6)
	Mean (SD)	0.0 (0.2)	0.1 (0.2)	0.8 (0.4)	1.3 (4.2)
***65–74*** *(* *N = 318)*	N (%)	1 (3.1)	33 (12.0)	248 (12.5)	429 (13.9)
	Mean (SD)	0 (0.1)	0.1 (0.4)	0.8 (0.4)	1.4 (5.5)
***≥ 75*** *(* *N = 711)*	Total	0	74 (26.9)	616 (31.1)	966 (31.2)
	Mean (SD)	0 (0)	0.1 (0.5)	0.9 (0.4)	1.4 (5.0)
***Risk groups***					
***Not at risk*** *(* *N = 1,240)*	N (%)	23 (71.9)	121 (44.0)	1,071 (54.1)	1,309 (42.3)
	Mean (SD)	0.0 (0.1)	0.1 (0.4)	0.9 (0.5)	1.1 (4.5)
***1 comorbidity*** *(* *N = 558)*	N (%)	7 (21.9)	92 (33.5)	456 (23.0)	978 (31.6)
	Mean (SD)	0.0 (0.1)	0.2 (0.6)	0.8 (0.4)	1.8 (5.5)
***2 + comorbidities*** *(* *N = 535)*	N (%)	2 (6.3)	62 (22.6)	453 (22.9)	809 (26.1)
	Mean (SD)	0 (0.1)	0.1 (0.4)	0.9 (0.4)	1.6 (5)

* Evaluated during the follow-up

** from admission date to one week after discharge date

^1^ Does not include day hospitals (*n* = 32)

^2^ Includes:

-ER accesses associated to a diagnosis of influenza

-ER accesses for all reasons that are followed by a hospitalization associated to a diagnosis of influenza within 2 days

### Healthcare costs associated with HRU in patients hospitalized with influenza

Table 4 shows the healthcare resource utilization (HRU), in terms of hospitalizations, day hospitals, outpatient visits, ER access laboratory tests and exams, prescribed drugs, and related direct costs per patient, overall, by age categories and risk groups for the entire study period. Overall, the mean (SD) cost-per-patient was € 4,185 (7,684) and was mainly attributable to hospitalization (€ 4,007, [7,620]). The highest median (Q3-Q1) costs were reported for adult individuals aged 65–74 (€ 3,030, [(4,742-2,250)]) and ≥ 75 (€ 3,230, [4,066 − 2,250]), p-value < 0.001, and were due to hospitalization (€ 2,679, [4,461-2,239] and € 3,096, [3,893-2,239] respectively). Direct overall healthcare costs were higher in patients at risk than in patients not at risk. The median (Q3-Q1) per patients’ cost was € 2,734 (4,098 − 1,866) for individuals with at least one comorbidity whilst it was € 2,257 (3,374-1,290) for patients not at risk and was mainly due to hospitalization. On average, costs were higher for patients with multiple comorbidities compared to patients with one comorbidity (Table 4). Age and risk-related patterns were observed also for outpatient visits. On average, costs were higher in individuals at extreme age categories, i.e., aged 0–17 (€ 4.3 [16.4]) and ≥ 75 (€ 3.5 [28.4]) and in individuals with at least one comorbidity (€4.6 [24.6]) compared to individuals not at risk (€ 2.6 [10.6]), p-value < 0.05. ER accesses, tests and lab exams and treatments have a minor impact on the overall economic burden, compared to hospitalization. Although the distribution of the associated costs was heterogeneous across age-categories and high costs were observed also in intermediate age groups, higher costs were recorded in patients at risk than in patients not at risk.

**Table 4:** Economic burden of HRU associated to influenza, by age categories and risk groups

Age categories (years)	Statistics	Total costs	Hospitalization^1^ *	Day Hospital*		Outpatient visits**	ER accesses^2^ *	Tests and lab exams**	Treatments**	
*All ages*	N	2,333	2,337	32		275	1,639	3,096	6,889	
	Total	9,765,439.6	9,348,715.1	5,868.9		7,802.0	164,808.7	72,215.9	166,029.0	
	Mean (SD)	4,185.8 (7,684.1)	4,007.2 (7,620.3)	2.5 (21.9)		3.3 (19.0)	70.6 (165.9)	30.9 (234.4)	71.2 (533.2)	
	Median (IQR)	2,534.8 (3,836.2-1,676.0)	2,445.0 (3,669.7-1,504.0)	0 (0–0)		0 (0–0)	0 (20.7-0)	0 (0–0)	0 (30.0 − 0)	
*0–17*	N (%)	596 (25.6)	598 (25.6)	10 (31.3)		86 (31.3)	337 (20.6)	595 (19.2)	476 (6.9)	
	Total	1,323,870.4	1,284,961.7	1,779.7		2,575.8	20,012.1	5,076.9	9,464.2	
	Mean (SD)	2,221.3 (2,258.9)	2,156.0 (2,232.7)	3.0 (23.5)		4.3 (16.4)	33.6 (78.4)	8.5 (45.7)	15.9 (105.0)	
	Median (IQR)	1,907.8 (2,704.0-880.1)	1,866 (2,704.0–808.5)	0 (0–0)		0 (0–0)	0 (20.7-0)	0 (0–0)	0 (0–0)	
*18–49*	N (%)	378 (16.2)	372 (15.9)	12 (37.5)		50 (18.2)	260 (15.9)	582 (18.8)	622 (9.0)	
	Total	1,731,772.3	1,662,746.3	2,291.4		1,225.1	30,644.5	7,921.1	26,943.9	
	Mean (SD)	4,581.4 (9,508.0)	4,398.8 (9,413.2)	6.1 (34.5)		3.2 (11.9)	81.1 (195.3)	21 (87.6)	71.3 (597.9)	
	Median (IQR)	2,250.0 (3,353.1-1,247.0)	2,184.0 (3,215.0–1,222.1)	0 (0–0)		0 (0–0)	0 (0–0)	0 (0–0)	0 (14.1-0)	
*50–59*	N (%)	218 (9.3)	218 (9.3)	6 (18.8)		26 (9.5)	148 (9.0)	383 (12.4)	746 (10.8)	
	Total	1,426,080.2	1,351,660.8	1,004.8		586	21,465.5	14,519.7	36,843.4	
	Mean (SD)	6,541.7 (13,069.8)	6,200.3 (13,035.3)	4.6 (28.0)		2.7 (8.2)	98.5 (211.1)	66.6 (373.5)	169.0 (1,081.0)	
	Median (IQR)	2,989.0 (4,550.0–2,250.0)	2,518.0 (3,910.0–2,147.0)	0 (0–0)		0 (0–0)	0 (130.9-0)	0 (0–0)	0 (40.0 − 0)	
*60–64*	N (%)	112 (4.8)	109 (4.7)	3 (9.4)		6 (2.2)	86 (5.3)	141 (4.6)	359 (5.2)	
	Total	660,010.3	612,842.1	601		122.2	12,464.9	8,590.6	25,389.5	
	Mean (SD)	5,893.0 (9.187.7)	5,471.8 (9,002.8)	5.4 (33.7)		1.1 (4.7)	111.3 (188.3)	76.7(399.1)	226.7 (1,462.7)	
	Median (IQR)	2,742.3 (4,619.2-1,774.4)	2,518.0 (3,893.0–1,676.0)	0 (0–0)		0 (0–0)	0 (207.2-0)	0 (0–0)	0 (40.9-0)	
*65–74*	N (%)	318 (13.6)	324 (13.9)	1 (3.1)		33 (12.0)	231 (14.1)	429 (13.9)	1,375.0 (19.9)	
	Total	1,846,616.8	1,785,957.8	192		819.2	22,707.5	12,499.7	24,440.6	
	Mean (SD)	5,807.9 (10,672.3)	5,616.2 (10,631.1)	0.6 (10.8)		2.6 (10.9)	71.4 (160.7)	39.3 (231.0)	76.9 (196.6)	
	Median (IQR)	3,030.3 (4,742.4-2,250.0)	2,679.5 (4,461.9-2,239.2)	0 (0–0)		0 (0–0)	0 (0–0)	0 (0–0)	3.6 (58.5-0)	
*≥ 75*	N (%)	711 (30.5)	716 (30.6)	0 (0)		74 (26.9)	577 (35.2)	966 (31.2)	3.311 (48.1)	
	Total	2,777,089.6	2,650,546.4	0		2.473.8	5,7514.2	2,3607.8	42,947.5	
	Mean (SD)	3,905.9 (4,319.2)	3,727.9 (4,292.3)	0 (0)		3.5 (28.4)	80.9 (180.1)	33.2 (286.3)	60.4 (127.6)	
	Median (IQR)	3,230.4 (4,066.4-2,250.0)	3,096 (3,893.0–2,239.2)	0 (0–0)		0 (0–0)	0 (0–0)	0 (0–0)	7.15 (71.6-0)	
**Risk group**										
*Not at risk*	N (%)	1,240.0 (53.2)	1,239.0 (53.0)	23 (71.9)		121 (44.0)	793 (48.4)	1,309.0 (42.3)	2,140.0 (31.1)	
	Total	4,604,207.4	4,459,107.4	4,214.6		3,209.8	77,895.0	15,984.7	43,796.0	
	Mean (SD)	3,713.1 (7,810.5)	3,596.1 (7,765.9)	3.4 (25.4)		2.3 (10.6)	62.8 (154.4)	12.9 (71.6)	35.3 (433.6)	
	Median (IQR)	2,257.0 (3,374.6-1,290.8)	2,250.0 (3,282.1-1,247.0)	0 (0–0)		0 (0–0)	0 (10.3-0)	0 (0–0)	0 (10 − 0)	
*1 comorbidity*	N (%)	558 (23.9)	558 (23.9)	7 (21.9)		92 (33.5)	422 (25.8)	978 (31.6)	2.072 (30.1)	
	Total	2,677,458.0	2,539,891.8	1,264.4		2,590.5	50,674.7	36,049.0	46,987.6	
	Mean (SD)	4,798.3 (8,181.3)	4,551.8 (8,121.1)	2.3 (20.8)		4.6 (24.6)	90.8 (195.4)	64.6 (401.9)	84.2 (302.6)	
	Median (IQR)	2,734.3 (4,098.0–1,866.0)	2,518.0 (3,893.0–1,676.0)	0 (0–0)		0 (0–0)	0 (104.3-0)	0 (0–0)	3.8 (50.0 − 0)	
*2 + comorbidities*	N (%)	535 (22.9)	540 (23.1)	2 (6.3)		62 (22.6)	424 (25.9)	809 (26.1)	2,677.0 (38.9)	
	Total	2,483,774.2	2,349,715.9	390		2,001.7	36,239.0	20,182.1	7,5245.4	
	Mean (SD)	4,642.6 (6,735.8)	4,392.0 (6,634.2)	0.7 (12.1)		3.7 (26.2)	67.7 (156.1)	37.7 (239.9)	140.7 (837.7)	
	Median (IQR)	3,166.6 (4,187.5-2,250.0)	2,868.0 (3,893.0–2,250.0)	0 (0–0)		0 (0–0)	0 (10.3-0)	0 (0–0)	7 (94 − 0)	

All costs are reported in Euros (€).

* Evaluated during the follow-up

** from admission date to one week after discharge date

^1^ Does not include day hospitals (*n* = 32)

^2^ Includes:

-ER accesses associated to a diagnosis of influenza

-ER accesses for all reasons that are followed by a hospitalization associated to a diagnosis of influenza within 2 days

### The clinical burden of influenza-related complications

**Fig. 3:**
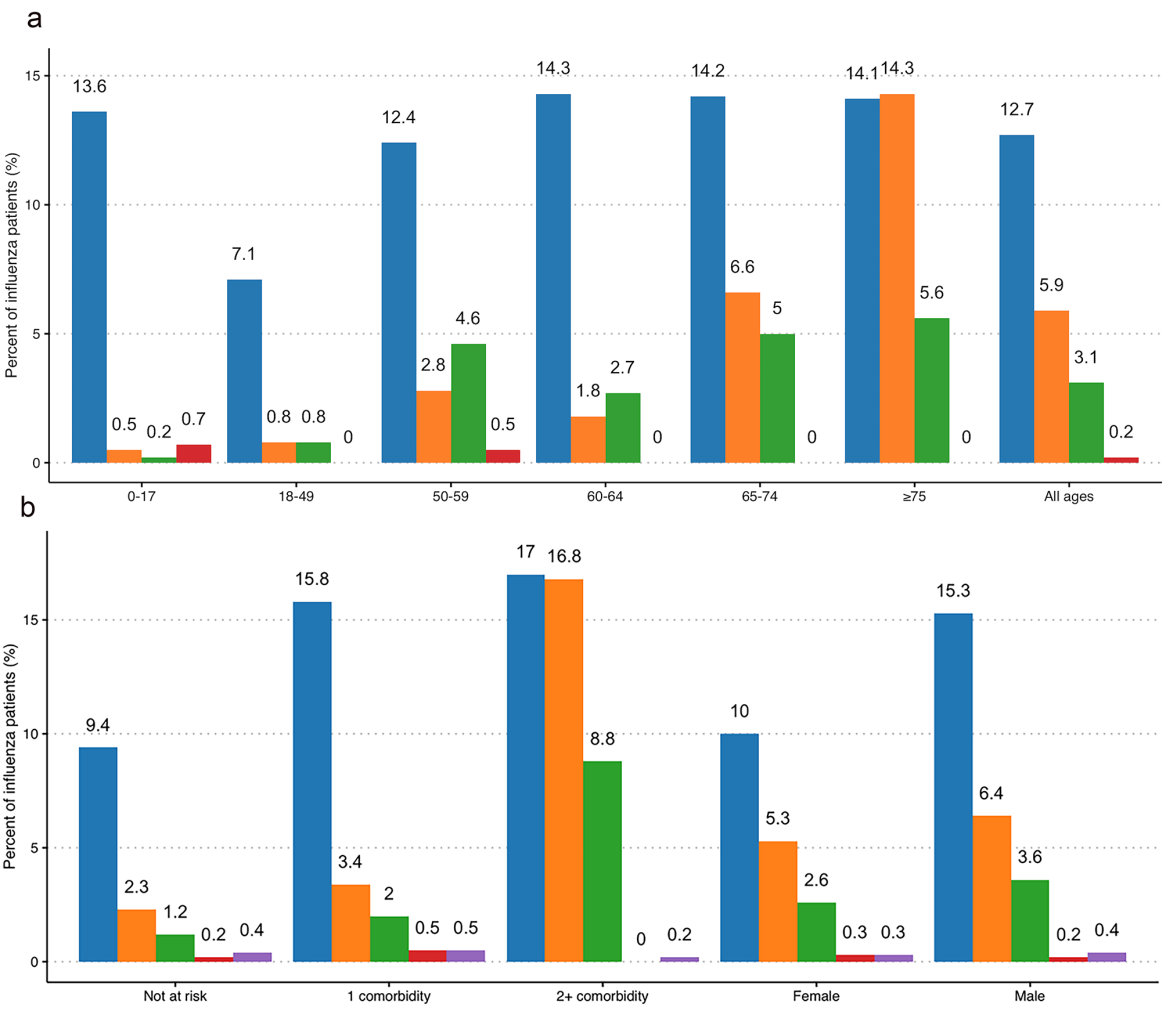
Distribution of complications across influenza patients by age categories (a), risk group and sex (b) **(a)**

Figure 3a and b show the rate of influenza related complications during 6-months follow-up stratified by age categories, risk group and sex. The prevalence and type of complication varied depending on the age group and disease type. Cardiovascular complications were mostly observed in adults. Patients aged ≥ 75 years (*N* = 711) showed the highest percentage of cardiovascular disorders (*n* = 102, 14.4%, 95% CI, 11.8–17.0), p-value < 0.001. Accordingly, also individuals at risk showed a high prevalence of cardiovascular diseases (≥ 2 comorbidities, *n* = 90, 16.8%, *N* = 535, 95% CI, 13.7–20.0). Respiratory complications were common across all age categories. Overall, they required hospital treatment or ER access in 12.7% (*n* = 296, 95% CI, 11.3–14.0) of the patients in the study cohort (*N* = 2,333). Their prevalence was high (p-value < 0.001) in patients at risk but even higher in patients with ≥ 2 comorbidities (*N* = 91, 17%) than in those with one comorbidity (*N* = 88, 15.8%) or not at risk (*N* = 117, 9.4%). It was higher in males (*n* = 182, 15.3%) than in females (*N* = 114, 10.0%), p-value < 0.001. Although the occurrence of respiratory complications was the lowest for patients aged 18–49 compared to the other age categories, it was the most frequently observed for those patients. Few diabetic complications were recorded (3.1% overall). They were mainly observed in adults aged ≥ 50 and in patients with two comorbidities (*n* = 47, 8.9%, 95% CI, 6.4–11.2). Neurological and co- and secondary infections were infrequent or even absent.

### In-hospital and overall mortality following hospitalization due to influenza

Table 5 shows in-hospital mortality due to all causes following hospitalization due to influenza overall and by epidemic season stratified by age categories and risk groups. The overall mortality during the follow-up is also reported. The age-related mortality was observed for the in-hospital deaths of influenza patients, due to all causes. Overall, mortality was higher in adults ≥ 65 years than in young individuals. The highest numbers of deaths were recorded among patients aged ≥ 75 (*N* = 34, *N* = 72, 47.2%) and among patients at risk (*N* = 46, *N* = 72, 63.9%) compared to patients not at risk (*N* = 26, *N* = 72, 36.1%). A total of 196 (8.4%, 95% CI, 7.3–10.0) patients of the entire cohort (*N* = 2,333) died during the 6-months follow-up period. Around 56.1% (*N* = 110, 95% CI, 49.2–63.1) of them, were ≥ 75. Eight patients died in the age group 0–17 (4.1%). Higher mortality in adults ≥ 65 years and in patients at risk was consistent across all epidemic seasons and reflected their relative severity. The seasons 2014–2015 (*n* = 19), 2016–2017 (*n* = 20) and 2017–2018 (*n* = 19) were the ones with the highest number of deaths, of which 78.9% (*n* = 15), 70.0% (*n* = 14) and 52.6% (*n* = 10) respectively occurred among individuals aged ≥ 65 years.

**Table 5:** Overall and in-hospital mortality due to all causes following hospitalizations due to influenza

		Age Categories						Risk Groups		
	All ages	0–17	18–49	50–59	60–64	65–74	≥ 75	Not at risk	1 comorbidity	2 + comorbidities
All causes in-hospital mortality (hospitalizations due to influenza) *, N (%)	72	4	6	6	7	15	34	26	24	22
*n, %*										
*2014-15*	19 (26.4)	1 (25.0)	.	2 (33.3)	1 (14.3)	4 (26.7)	11 (32.4)	5 (19.2)	6 (25.0)	8 (36.4)
*2015-16*	1 (1.4)	.	.	.	.	.	1 (2.9)	1 (3.9)	.	.
*2016-17*	20 (27.8)	.	2 (33.3)	.	4 (57.1)	4 (26.7)	10 (29.4)	5 (19.2)	7 (29.2)	8 (36.4)
*2017-18*	19 (26.4)	3 (75.0)	3 (48.0)	3 (48.0)	.	3 (20.0)	7 (20.6)	12 (46.2)	4 (16.7)	3 (13.6)
*2018-19*	12 (16.7)	.	1 (16.7)	1 (16.7)	2 (28.6)	4 (26.7)	4 (11.8)	3 (11.5)	6 (25.0)	3 (13.6)
**Mortality during 6-month-period of follow-up** **(** *N = 2,333)*	196 (8.4)	8 (1.3)	14 (3.7)	9 (4.1)	18 (16.1)	37 (11.6)	110 (15.5)	58 (4.7)	66 (11.9)	72 (13.4)

All results are reported as absolute numbers (N) and percentages (%), in parenthesis. These last are calculated, for each influenza season, over the number of deaths by age category and risk groups reported for all cause in-hospital mortality, while they refer to the total number of patients reported in Table 1 for each age category, for the mortality during 6-months follow-up.

*Mortality was recorded both as in-hospital mortality and mortality during 6-month follow-up period.

## Discussion

The aim of this retrospective observational study was to investigate the clinical and economic burden of patients with severe seasonal influenza in Italy across five epidemiological influenza seasons (2014–2019). Administrative data from 4 LHUs were used to describe hospitalization, mortality, and resource utilization, including direct costs, of a cohort of 2,333 patients hospitalized with flu. The presence of comorbidities at the admission and the occurrence of complications after discharge, were also considered for a more detailed description of the healthcare burden of the disease.

Overall, in this study, the number of females and males hospitalized with severe disease was comparable although with a prevalence of males (51%) compared to females (49%). The mean age of study participants at admission was 50 years.

Of the entire cohort, individuals aged 17 years (25.6%) or less, and adults aged ≥ 65 (56.0%) were the most affected by serious illness, consistently with what observed in other high-income countries [25–30]. The two groups represent vulnerable individuals at higher risk than individuals of other age categories, because of immunosenescence or a weaker immune system [31]. A history of at least one comorbidity before the first hospitalization for flu was observed in almost half (46%) of the patients. The most frequently observed were elderly illnesses, like cardiovascular and metabolic diseases, chronic medical conditions like diabetes, disorders of pulmonary circulation and neoplasms. Together with older age, they represent risk factors of severe influenza that require hospitalization, and disease-related complications particularly in older adults [29, 32, 33].

Consistently with age-related fragility and co-existence of additional medical conditions, the mean hospitalization rate over the 5 influenza seasons, was significantly higher in older patients (i.e., ≥ 65 years old) in line with what observed in other countries [34–36]. From our data, rates of hospitalization between males and females were comparable. The overall number of admissions was higher for patients with comorbidities than for patients without them.

Variability in influenza-associated hospitalization rates was observed throughout the epidemic seasons and was consistent with national trends of influenza severity and intensity [37]. The 2015–2016 influenza season was recorded as the less severe in Italy, in Europe [38] and US [39], while seasons 2014–2015, 2017–2018 and 2018–2019 were reported as the ones with the highest influenza activity. Consistently, we observed a decrease in the hospitalization rate in the 2015–2016 season and an increase in the remaining seasons, thus reflecting the trend observed in national influenza surveillance data based on cases reported by general practitioners. This, highlights that although the patients included in this study represent a sample of the entire Italian population, our cohort from the 4 LHUs was effective in representing a broader national scenario and providing a robust description of the influenza burden in Italy.

When hospitalized, patients with severe influenza stayed 8 days, on average. This duration was longer for adults aged ≥ 65 years and for patients at risk (11 days). Prolonged hospitalization for severe adult patients and for those with additional chronic conditions (about 9 days) has also been observed in other countries [40] although with a certain degree of variability due to different national healthcare frameworks and cohort heterogeneity [41, 42]. More than 50% of admissions were managed by the general medicine and pediatric departments. Few readmissions occurred in the 6 months following discharge, most of which involved patients in extreme age categories. From our data, re-hospitalization does not represent a significant healthcare burden.

Mortality of influenza patients in our cohort was low. Few patients (*N* = 72; 3% of 2,333) died of any cause during hospitalization for influenza or within 6 months after discharge. Most of them (47.2%) were elderly (≥ 75) and with chronic diseases (63%). As recently shown [19, 43, 44], patients accumulating comorbidities and of older age, have a higher mortality risk than those without. This highlights an interplay between influenza infection and pre-existing medical conditions and the role of the latter as predictors of worse outcomes.

As discussed by *Macias et al.* [15]. , complications directly resulting from the viral infection, or the exacerbation of underlying conditions represent a secondary burden of influenza with a considerable impact on healthcare and economic systems. In a Spanish study, approximately 12% of severe ill patients with a confirmed influenza diagnosis, experienced at least one acute cardiovascular event in the presence of underlying chronic conditions [45]. Patients in our cohort also developed serious complications that required hospital care or ER access. Cardiovascular and chronic diseases such as diabetes, were mainly observed in adults while respiratory complications were also common in young patients in line with what has been reported by *Macias et al.* [15]. , and in other studies [46–48] from different countries.

Hospitalization was the major HRU together with emergency room access which occurred before 84% of all admissions and was more frequent among young and elderly individuals. Few tests or laboratory exams (~ 1) were performed, on average during the hospital stay. An increase was observed in patients at risk, that required more investigations. Similarly, outpatient visits were more frequent among young children, individuals with underlying medical conditions, and the elderly. On average, the total costs were considerable and amounted to €9.7 million for the entire study period. Hospitalization costs accounted for almost 95% of the total direct costs of influenza, with €9.3 million. Of these, €2.6 million were attributed to elderly (≥ 75 years) and €2.6 million to patients at risk. The same was observed, on average, for outpatient visits whose impact on the economic burden weas greater for individuals in extreme age categories (i.e., 0–17 and ≥ 65) and with underlying comorbidities. Overall, the average cost (SD) per-inpatient stay was €4,007 (€ 7,620) for all epidemic seasons. Similarly, a recent study [49] on Turkish population aged > 18 years referring to the epidemic season 2018–2019 showed daily hospital costs equal to 3,274 USD in the influenza-positive group (*N* = 55), a higher value than non-influenza patients (*N* = 137; 2,765 USD) but also patients admitted with other respiratory infections but not influenza (*N* = 70; 3,103 USD). Taken together, our data highlight the significant clinical burden of seasonal influenza mainly on vulnerable individuals, and the high economic impact on the Italian healthcare system. Immunization with influenza vaccine [50] in addition with non-pharmaceutical public health measures [51, 52] may reduce the risk of getting severe illness, reduce the burden of its complications and the costs to the national government.

### Limitations of the study

This study is based on data from four LHUs in Italy. Although the results are interesting from a public health perspective, they may not be fully generalized as the LHUs may not be representative of the whole national medical practice. The selection of geographically distributed mixture of heterogeneous centres, allowed to reduce the limited representativeness of the results but larger studies are needed to confirm our findings. Furthermore, as this is a retrospective real-world longitudinal study on Administrative Databases, data are not standardized across participating centres, and might be incomplete or differentially reported. Selection bias, i.e., the loss of patients using health services in a different region, and information bias, i.e., treatments in private hospitals for which reimbursement flow is not available, may also have an impact on the robustness of the results. Furthermore, patients were not classified according to vaccination status due to the lack of data on vaccine administration in Administrative Databases in Italy. Therefore, the impact of vaccination on clinical and economic burden cannot be directly assessed in the present study. Further data from vaccination centres, pharmacies and general practitioners are needed to investigate and quantify the effect of immunization on the hospital burden of influenza. Finally, the actual number of patients hospitalized with influenza might be underestimated due to the incorrect coding of flu diagnosis frequently observed in the SDO and due to the low number of diagnostic tests.

## Conclusions

This is the first nationwide study that analyse the clinical and economic burden of hospitalized patients in Italy, with severe influenza during 5 epidemic seasons (2014–2019) and over a wide range of individual ages, from claims data. Our results show that age and underlying medical conditions increase the risk to develop severe illness that requires hospitalization. A weak and underdeveloped immune system in infants and young children and, conversely immunosenescence resulting from aging in adults and particularly in the elderly are the main determinants of the observed increase in the incidence of serious viral infection among these vulnerable individuals. Complications, mainly cardiac and respiratory, directly resulting from influenza or due to exacerbation of pre-existing comorbidities also worsen the clinical outcome of them and give rise to an additional influenza burden with an economic impact mainly due to required hospitalization or ER access and prolonged hospital stay. Although with limitations, mainly due to poor diagnosis and coding practices, our study broadly describes the Italian clinical burden of severe influenza reflecting the national trends reported by surveillance systems and provides data on the economic burden on the national government. Our results should be used to inform public health decision-making.

### Electronic supplementary material

Below is the link to the electronic supplementary material.

Supplementary Material 1

## Data Availability

Data can be made available on request on a case-by-case basis and depending on legal/privacy regulations.

## Abbreviations

ADs: Administrative Databases
LHUs: Local Health Units
ID: Index date
IQR: Interquartile Range
AD: Administrative Data
ATS: Agenzia di Tutela della Salute
ULSS: Unità Locale Socio–Sanitaria
ASL: Azienda Sanitaria Locale
SDO: Scheda di Dimissione Ospedaliera
DRG: Diagnosis-Related Group
ER: Emergency Room
ATC: Anatomical-Therapeutic-Chemical
EC: Ethics Committee
HRU: Healthcare Resource Utilization
INHS: National Health Service
SD: Standard Deviation
LTCF: Long-term Care Facilities
CIs: Confidence Intervals
