# Supplementary material for: The Hospital Burden of Flu in Italy: a retrospective study on administrative data from season 2014–2015 to 2018–2019
Source: BMC Infect Dis. 2024 Jun 8;24:572. doi: 10.1186/s12879-024-09446-2 (PMC11162570; doi:10.1186/s12879-024-09446-2)
Supplement: Supplementary file 1 — Supplementary Material 1 [file 12879_2024_9446_MOESM1_ESM.docx]

**The Hospital Burden of Flu in Italy: A Retrospective Study on Administrative Data from Season 2014-2015 to 2018-2019**

Riccardo Cipelli^a*^, Serena Falato^a^, Eleonora Lusito^a^, Giovanni Maifredi^b^, Michele Montedoro^c^, Paola Valpondi^d^, Alberto Zucchi^e^, Maria Vittoria Azzi^f^, Laura Zanetta^f^, Maria Rosaria Gualano^g^, Entela Xoxi^h^, Paola Giovanna Marchisio ^i, m, §^ and Silvana Castaldi ^l, m, §^

^a^IQVIA Solutions Italy Srl, Milan, Italy

^b^SS Epidemiologia, Agenzia di Tutela della Salute di Brescia, Italy

^c^UVARP ASL Foggia, San Severo c/o Ospedale, Italy

^d^ULSS 8 Berica, Vicenza, Italy

^e^UOC Servizio Epidemiologia presso ATS di Bergamo, Italy

^f^Sanofi SpA, Milan, Italy

^g^Saint Camillus International University of Health Sciences, Rome, Italy

^h^Università Cattolica del Sacro Cuore, Alta Scuola di Economia e Management dei Sistemi Sanitari (ALTEMS), Rome, Italy

^i^Dipartimento di Fisiopatologia Medico-Chirurgica e dei Trapianti, University of Milan, Italy

^l^ Department of Biomedical Sciences for Health, University of Milan, Italy

^m^Fondazione IRCCS Ca' Granda Ospedale Maggiore Policlinico, Milan, Italy

^§^ Paola Giovanna Marchisio and Castaldi Silvana contributed equally to this work.

^*^ Correspondence: Riccardo Cipelli, [riccardo.cipelli@iqvia.com](mailto:riccardo.cipelli@iqvia.com). IQVIA Solutions; Via Fabio Filzi 29; 20124 Milan; Italy.

**Supplementary Material**

**Table S1.** Hospitalization rates overall and by influenza seasons, stratified by age categories and sex

|  | **Age categories** | | | | | | | **Sex** | |  |
| --- | --- | --- | --- | --- | --- | --- | --- | --- | --- | --- |
|  |  |  |  |  |  |  |  |  |  |  |
| **Estimated hospitalization rates*, n** | **All ages** | **0 - 17** | **18 - 49** | **50 - 59** | **60 - 64** | **65 - 74** | **≥75** | **Male** | **Female** |  |
|  |  |  |  |  |  |  |  |  |  |  |
| *2014-15* | 12.8 | 15.4 | 5.5 | 9.3 | 12.7 | 19.6 | 33.2 | 13.2 | 12.3 |  |
| *2015-16* | 6.7 | 14.1 | 2.8 | 3.0 | 5.2 | 6.3 | 16.6 | 6.4 | 7.1 |  |
| *2016-17* | 12.9 | 11.1 | 4.2 | 5.6 | 12.7 | 17.4 | 53.3 | 12.6 | 13.1 |  |
| *2017-18* | 16.0 | 30.4 | 6.7 | 10.1 | 9.4 | 22.1 | 33.2 | 16.3 | 15.6 |  |
| *2018-19* | 17.3 | 26.5 | 7.0 | 9.9 | 11.3 | 19.9 | 51.4 | 18.8 | 15.7 |  |
| *Out of season* | 4.5 | 9.0 | 2.6 | 3.9 | 1.4 | 3.8 | 7.6 | 5.4 | 3.7 |  |
| *Mean hospitalization rates*  *over 5 years* | 13.1 | 19.5 | 5.3 | 7.6 | 10.3 | 17.1 | 37.6 | 13.5 | 12.8 |  |

*Estimated hospitalization rates (per 100.000 inhabitants) were calculated using aggregated data published by the LHUs on age and sex [Ministero della Salute - Direzione Generale della digitalizzazione, del sistema informativo sanitario e della statistica - Ufficio di statistica; Fonti: ISTAT: Popolazione residente al 31 dicembre 2019]

**Table S2:** Ward occupation during the inpatient stay period

|  |  | **Age categories** | | | | | | **Risk groups** | | |
| --- | --- | --- | --- | --- | --- | --- | --- | --- | --- | --- |
| **Department** | **All ages** | **0-17** | **18-49** | **50-59** | **60-64** | **65-74** | **≥75** | **Not at risk** | **1 comorbidity** | **2+**  **comorbidity** |
|  | **(N = 2,333)** | **(N=596)** | **(N=378)** | **(N=218)** | **(N=112)** | **(N=318)** | **(N=711)** | **(N = 1,240)** | **(N = 558)** | **(N = 535)** |
|  |  |  |  |  |  |  |  |  |  |  |
| *General*  *Medicine* | 919 (39.4) | 7 (0.3) | 115 (4.9) | 83 (3.6) | 45 (1.9) | 162 (6.9) | 507 (21.7) | 359 (15.4) | 251 (10.8) | 309 (13.2) |
| *Pediatrics* | 520 (22.3) | 515 (22.1) | 5 (0.2) | . | . | . | . | 446 (19.1) | 59 (2.5) | 15 (0.6) |
| *Infectious and tropical diseases* | 268 (11.5) | . | 85 (3.6) | 53 (2.3) | 26 (1.1) | 39 (1.7) | 65 (2.8) | 140 (6.0) | 70 (3.0) | 58 (2.5) |
| *Pneumology* | 110 (4.7) | 1 (0.0) | 19 (0.8) | 16 (0.7) | 9 (0.4) | 27 (1.2) | 38 (1.6) | 44 (1.9) | 32 (1.4) | 34 (1.5) |
| *Obstetrics and Gynecology* | 68 (2.9) | . | 66 (2.8) | 1 (0.0) | 1 (0.0) | . | . | 25 (1.1) | 36 (1.5) | 7 (0.3) |
| *Cardiology* | 48 (2.1) | . | 8 (0.3) | 6 (0.3) | 1 (0.0) | 9 (0.4) | 24 (1.0) | 13 (0.6) | 6 (0.3) | 29 (1.2) |
| *Nephrology* | 48 (2.1) | . | 8 (0.3) | 13 (0.6) | 4 (0.2) | 10 (0.4) | 13 (0.6) | 9 (0.4) | 14 (0.6) | 25 (1.1) |
| *Hematology* | 47 (2.0) | . | 11 (0.5) | 13 (0.6) | 6 (0.3) | 12 (0.5) | 5 (0.2) | 7 (0.3) | 26 (1.1) | 14 (0.6) |
| *Neurology* | 45 (1.9) | . | 18 (0.8) | 6 (0.3) | 5 (0.2) | 6 (0.3) | 10 (0.4) | 30 (1.3) | 11 (0.5) | 4 (0.2) |
| *General surgery* | 40 (1.7) | 2 (0.1) | 15 (0.6) | 6 (0.3) | 2 (0.1) | 10 (0.4) | 5 (0.2) | 20 (0.9) | 14 (0.6) | 6 (0.3) |
| *ICU* | 177 (7.6) | 44 (1.9) | 34 (1.5) | 21 (0.9) | 15 (0.6) | 31 (1.3) | 32 (1.4) | 97 (4.2) | 40 (1.7) | 40 (1.7) |
| *Neonatal ICU* | 9 (0.4) | 9 (0.4) | . | . | . | . | . | 8 (0.3) | 1 (0.0) | . |

All results are reported as absolute numbers (N) and percentages (%), in parenthesis. These last are calculated over N = 2,333.

**Table S3.** Patient path after discharge

|  |  | **Age categories** | | | | | | **Risk groups** | | |
| --- | --- | --- | --- | --- | --- | --- | --- | --- | --- | --- |
| **Category** | **All ages** | **0-17** | **18-49** | **50-59** | **60-64** | **65-74** | **≥75** | **Not at risk** | **1 comorbidity** | **2+ comorbidity** |
|  | **(N=2,333)** | **(N=596)** | **(N=378)** | **(N=218)** | **(N=112)** | **(N=318)** | **(N=711)** | **(N=1,240)** | **(N=558)** | **(N=535)** |
|  |  |  |  |  |  |  |  |  |  |  |
| *Patient that went home after being discharged* | 2,122 (91.0) | 588 (27.7) | 366 (17.3) | 194 (9.1) | 94 (4.4) | 277 (13.1) | 603 (28.4) | 1,154 (54.4) | 508 (23.9) | 460 (21.7) |
| *Patients transferred to another hospital/service* | 127 (5.4) | 4 (3.2) | 10 (7.9) | 20 (15.6) | 10 (7.9) | 26 (20.5) | 57 (44.9) | 54 (42.5) | 24 (18.9) | 49 (38.6) |
| *Patients transferred to LTCF* | 28 (1.2) | 2 (7.1) | 1 (3.6) | 2 (7.1) | 0 (0.0) | 2 (7.1) | 21 (75.0) | 16 (57.1) | 4 (14.3) | 8 (28.6) |

All results are reported as absolute numbers (N) and percentages (%), in parenthesis. These last are calculated, for each age category and risk group over the total number of patients of all ages class of the relative category.
